# Volatile Organic Compounds in Finnish Office Environments in 2010–2019 and Their Relevance to Adverse Health Effects

**DOI:** 10.3390/ijerph19074411

**Published:** 2022-04-06

**Authors:** Kaisa Wallenius, Hanna Hovi, Jouko Remes, Selma Mahiout, Tuula Liukkonen

**Affiliations:** Finnish Institute of Occupational Health, P.O. Box 40, FI-00032 Työterveyslaitos, Finland; hanna.hovi@ttl.fi (H.H.); jouko.remes@ttl.fi (J.R.); selma.mahiout@ttl.fi (S.M.); tuula.liukkonen@ttl.fi (T.L.)

**Keywords:** indoor air quality, VOC, formaldehyde, office, health risk, trend

## Abstract

We gathered recent (2010–2019) data on the VOC and formaldehyde levels in Finnish non-industrial indoor work environments. The data comprised 9789 VOC and 1711 formaldehyde samples collected from the indoor air of offices, schools, kindergartens, and healthcare offices. We assessed the health risks by comparing the measured concentrations to the health-based RW I/II and EU-LCI reference values. The concentrations of individual VOCs and formaldehyde in these work environments were generally very low and posed no health risks. Total VOC concentration (TVOC) as well as concentrations of several individual compounds, including aromatic compounds, alkanes, 2-ethyl-1-hexanol, and formaldehyde, showed clearly decreasing trends. In contrast, several aldehydes, acids, and a few other compounds showed increasing trends. However, the increasing trends did not seem to affect the higher ends of the distributions, as the 95th percentile values remained fairly stable or decreased over the years. The VOC patterns in the environments of the offices, schools, kindergartens, and healthcare offices varied, probably reflecting the differences in typical activities and the use of materials. However, we do not expect these differences to be relevant to health outcomes.

## 1. Introduction

Volatile organic compounds (VOCs) are a versatile group of chemical compounds that are present both indoors and outdoors. Indoor air has a greater variety of different VOCs than outdoor air, and the concentrations of these compounds are typically higher indoors than outdoors [1,2,3,4]. The differences in the composition of indoor and outdoor air derive from greater human impact, a larger surface area to volume ratio, and lower levels of light and oxidising agents indoors [5]. Typical groups of the organic compounds present in indoor air are alkanes, terpenes, aromatic hydrocarbons, and aldehydes [4,6]. 

The spatial and temporal variation of volatile compounds in indoor environments is great. This variation is governed by both natural and man-made sources of emissions both indoors and outdoors. Indoor emission sources include building and interior materials, technical systems and equipment, buildings’ occupants, and a large variety of chemicals, products, and materials used and produced by occupants in different activities. The main outdoor emission sources are traffic and industrial processes. VOC levels are impacted by the seasons [3,7] and the geographic locations of building [6,7,8,9]. The long-term chemical composition of indoor environments changes as new technologies and materials are developed [10,11,12].

Concerns over possible health risks related to VOC emissions in the indoor air are prevalent. Many VOCs and formaldehyde are known to be hazardous and to pose health risks in high concentrations in, for example, industrial work environments. The same does not necessarily apply to non-industrial environments if the concentrations remain below levels that induce health hazards.

The aim of the study was to (1) compile recent measurement data on the levels of VOCs and formaldehyde in offices and similar non-industrial indoor work environments in Finland and (2) to evaluate the health relevance of these. We also extended the study to analyse trends during the study period (2010–2019) and to inspect the differences between the environments of offices, schools, kindergartens, and healthcare offices.

To analyse the VOCs, we used the definition and analytical procedure described in the ISO 16000-6:2011 standard, which defines VOCs as substances that elute between n-hexane and n-hexadecane under defined chromatographic conditions. To analyse formaldehyde, which belongs to very volatile organic compounds (VVOC), we used an ISO 16000-3:2011-based analytical procedure.

## 2. Materials and Methods

### 2.1. Description of Data

The data of this study comprised results from indoor air samples of VOCs and formaldehyde from Finnish offices and similar non-industrial work environments analysed at the laboratory of the Finnish Institute of Occupational Health (FIOH) between January 2010 and December 2019. The total number of samples to measure VOCs was 9789, collected from offices (3872 samples), schools (3583 samples), kindergartens (727 samples), and healthcare offices (1607 samples). Healthcare offices were office-like spaces in healthcare premises where patients were not treated. The total number of samples for measuring formaldehyde was 1711, collected from offices (521 samples), schools (938 samples), kindergartens (68 samples), and healthcare offices (184 samples). The yearly distributions of VOC and formaldehyde samples are presented in Table 1.

The sampling was performed by experts from a multitude of consultant engineering offices (customers of FIOH’s analytical laboratory) as part of indoor air services, without a systematic monitoring programme. The samples were collected from occupied buildings during all seasons and from different parts of Finland. Winter (December, January, February) and spring (March, April, May) seasons accounted for approximately 60% (30% each) of all the VOC samples collected, whereas summer (June, July, August) and autumn (September, October, November) were less active sampling seasons with the shares of 15% summer and 24% autumn. Similar to VOCs, the seasonal distribution of formaldehyde samples was 32% winter, 27% spring, 17% summer, and 25% autumn.

The majority of the samples were collected from buildings with suspected indoor air-quality problems. In the buildings in which VOCs were measured, the suspected problems did not necessarily concern VOC pollution. In contrast, formaldehyde was measured in buildings in which the suspected problems specifically concerned formaldehyde. The laboratory did not record the age, the renovation status, or technical characteristics of the buildings, but we assume that the studied premises represent well the relatively homogenous Finnish building stock of public and office buildings built in 1960–2000. The vast majority of public and office buildings in Finland are equipped with mechanical supply and exhaust ventilation. The average number of samples per one sampling occasion (forming one laboratory assignment) was 3.1 for VOC (3167 sampling occasions) and 3.3 for formaldehyde (520 sampling occasions). The samples from single sampling occasions were mainly collected from one building but from different rooms. Thus, the number of sampling occasions gives a rough estimate of the number of sampled buildings.

### 2.2. Sampling and Analysing VOCs

The VOCs were sampled and analysed in accordance with the ISO 16000-6:2011 standard. The samples were collected in Tenax TA or Tenax TA/Carbograph 5 TD tubes from central room locations 1–1.5 m above floor level, mainly (99.5%) by active sampling (pump). Diffusive sampling was used for approximately 0.5% of the samples. We analysed the samples using thermal desorption and GC/MS. The compounds were identified by comparing them to pure reference substances and/or the Wiley or NIST mass spectral databases. FIOH’s laboratory has approximately 150 different compounds calibrated with their own response factors. Calibrated compounds cover most of the common compounds in indoor air. Some individual compounds that are not frequently identified or quantified from samples, and some mixtures, such as higher boiling alcohols or hydrocarbon mixtures, are quantitated as toluene equivalents because reference compounds are not available. All the individual compounds whose results are presented in this article were quantified with substance-specific responses. The total concentration of volatile organic compounds (TVOC) was determined for each sample as a toluene-equivalent concentration. TVOC was determined as the area in the chromatogram between the n-hexane and n-hexadecane, including both substances.

FIOH’s laboratory has ISO 17025 accreditation. The laboratory provided detailed sampling instructions and calibrated sampling equipment for the experts who performed sampling. The recommended sampling volume in active sampling was 7–12 dm^3^. Typical sampling volume was 9 dm^3^, which is equal to 45 or 90 min of sampling time. Diffusive sampling times were 2 to 4 weeks. The laboratory evaluated sampling volumes and times for every sample.

### 2.3. Sampling and Analysing Formaldehyde

Formaldehyde was sampled and analysed in accordance with the ISO 16000-3:2011 and the ISO 16000-4:2011 standards. The samples were collected in 2,4-dinitrophenylhydrazine (DNPH)-coated silica gel cartridges (Sep-Pak^®^ DNPH-Silica Plus Short Cartridge) using active sampling (91%) or in DNPH-coated passive samplers (UMEx 100 Passive Sampler for formaldehyde) using diffusive sampling (9%). The DNPH derivate of formaldehyde was desorbed using acetonitrile and then analysed with a high-performance liquid chromatography (HPLC) that contained a UV detector. Formaldehyde was identified and quantitated with reference compounds.

The laboratory provided detailed sampling instructions and calibrated sampling equipment for the experts who performed sampling. Recommended sampling volume in active sampling was 100 dm^3^, which is equal to 100 min of sampling time. Diffusive sampling time was 24 h. The laboratory evaluated sampling volumes and times for every sample.

### 2.4. Statistical Methods

First, we calculated the frequencies of the different analytes. Frequency indicates the percentage of samples whose results exceed the limit of quantitation (LOQ). To describe the distribution of the variables, we calculated the 50th percentile (median, Md), 90th percentile (P90), 95th percentile (P95), and 99th percentile (P99), first covering all the samples and then separately for different indoor environments and different years (2010–2019). For percentiles, we calculated the 95% confidence intervals based on normal distribution.

The association between the frequency of the different measures in the different types of environments was analysed using log-binomial regression analysis. The office was used as a reference group. We dichotomised the values such that <LOQ values were 0, and ≥LOQ values were 1. In log-binomial regression analyses, we compared percentages (value 1) separately school/kindergarten/healthcare offices to offices’ percentages. We calculated the crude risk ratios (RR) and their 95% confidence intervals and made no adjustments.

We analysed the frequency and concentration trends of each compound. The frequency trend was tested using Somer’s D test, in which the input data were binomial (measurement result either ≥LOQ or <LOQ). The concentration trend was tested using Somer’s D test, in which the input data comprised all the measured concentration values. Here, measurement results that were <LOQ were replaced by a concentration value of LOQ/2. Statistically significant trends were evaluated using the Z value. This statistic is used to test the null hypothesis, according to which no trend exists. A positive Z indicates an increasing trend in a time-series, while a negative Z indicates a decreasing trend. We used the SPSS and SAS statistical software packages for our calculations.

### 2.5. Health Risk Assessment

Health-based RW I/II and EU-LCI values were used to evaluate the possible health risks of VOCs and formaldehyde at the concentration levels measured. Both reference values have been set by experts using epidemiological and toxicological data. We primarily used the RW I/II values. If the RW values were not available, we used the European EU-LCI values if they were available.

The German RW I and II values [13,14] are reference values for indoor air-quality assessment that are set to cover the entire population and long-term exposure. In Germany, they are used in assessment of indoor air quality in homes, offices, schools, and other public spaces. The RW II values represent concentration levels that may cause adverse health effects for individuals who are sensitive due to their health status, for example, if they are exposed to these concentration levels for extended periods of time. The RW I values represent concentration levels which, in the light of current knowledge, do not cause adverse health effects even in cases of lifetime exposure.

The EU-LCI values (LCI, Lowest Concentration of Interest) are intended for assessing the safety of construction products regarding the potential health risks posed by inhaling emissions from new products [15]. The values aim to prevent health risks and cover the lifetime exposure of the entire population. They therefore represent concentration levels that, in the light of current knowledge, are considered unlikely to cause adverse health effects even in the long term. However, the EU-LCI values are not intended for use as reference values for assessing indoor air quality; they are for assessing building material emissions under experimental conditions. Nonetheless, as they were derived using the principles of toxicological hazard assessments, we considered them adequate for use in our study as indicative reference values when RW values were not available.

Health risk assessment was performed by calculating risk characterisation ratios (RCRs; [16]) for the 42 most frequently detected VOCs. The calculations were made by dividing the 99th percentile (P99) of the measurements by the above-mentioned health-based reference values. The RCRs were considered to indicate a potential health risk if they exceeded 1.

## 3. Results

### 3.1. Statistics of Entire Ten-Year Data

Around 400 different analytes (single compounds or mixtures that cannot be separated by the measurement protocol) were detected and quantitated at least once in the ten-year VOC data, which consisted of 9789 air samples. Formaldehyde data collected and analysed using a different procedure from that used for VOC during the same study period consisted of 1711 air samples. The LOQ for different compounds ranged between 0.3 and 1 µg/m^3^. The number of VOCs that had a frequency of ≥10% was 42. Table 2a presents the descriptive statistics of these 42 VOCs and the TVOC sum variable. Table 2b presents the corresponding statistics for the formaldehyde measurements. In addition to the measurement data, Table 2 is supplemented with up-to-date health-based reference values (EU-LCI, RW I, and RW II) for respective compounds if available.

The median values of the 42 most common VOCs were mostly below the respective LOQ: only 12 compounds had a frequency greater than 50% (Table 2a). P90 values were typically only slightly above the respective LOQ: 0.5–3 µg/m^3^. The largest P90 value was observed for 1,2-propanediol, at 7 µg/m^3^. P95 values ranged from 0.6 µg/m^3^ (octane) to 13 µg/m^3^ (1,2-propanediol). P99 values ranged from 1 µg/m^3^ (octane, nonane) to 45 µg/m^3^ (1,2-propanediol). The maximum values were tenfold to hundredfold larger than the P99 values for most of the compounds. The maximum values ranged from 12 µg/m^3^ (octane) to 1110 µg/m^3^ (xylenes p, m).

None of the RCRs, representing possible health risks, exceeded 1 when the P99 values of the 42 common VOCs were compared with their respective RW I, RW II, and EU-LCI values; all P99 values were well below their respective health-based reference values (Table 2a). The highest RCR compared to the RW I values, 0.75, was found for 1,2-propanediol.

No RW I/II or EU-LCI values exist for the alkanes nonane, octane, and 2,2,4,6,6-pentamethylheptane. All their P99 values (1–6 µg/m^3^) remained notably below the EU-LCI value of heptane (15,000 µg/m^3^), which belongs to the same group of alkanes. Based on the similar chemical properties and health hazard profiles of all these four alkanes, we can assume that this reference value would probably be in the same range as their own EU-LCI values. No RW I/II or EU-LCI values are available for benzene, but the UBA derived risk-related guide values for carcinogenic substances in indoor air [13]. The risk-related value for benzene is 4.5 µg/m^3^ (preliminary value), which is above the P99 value of 3.0 µg/m^3^ [14].

The maximum values of several of the 42 most common VOCs exceeded their RW I values. The maximum values for xylenes, 1,2-propanediol, 2-(2-ethoxyethoxy)ethanol, and phenoxyethanol also exceeded the RW II and/or EU-LCI values. In addition, the maximum value of benzene exceeded its risk-related value.

The TVOC sum variable has no health-based reference values. The health relevance of TVOC can only be meaningfully evaluated if the components of the total concentration are known.

The formaldehyde results revealed a shape of distribution that was different from most of the VOCs (Table 2b). For formaldehyde, the gap between P99 and the maximum values was notably smaller, and the proportion of the results that fell below the LOQ was also smaller than that for most VOCs. The formaldehyde data represented buildings/indoor locations that indoor air specialists anticipated to be at risk of elevated formaldehyde concentrations. Therefore, it is likely that in this sample of buildings, formaldehyde occurred more frequently and in higher concentrations than among the normal population of this type of building. Still, the maximum concentration of formaldehyde measured was also below the respective health-based RWI and EU-LCI reference values.

The majority of all the VOCs detected during the ten year-study period were present in less than 10% of the samples. These infrequent VOCs represented a wide variety of different organic compounds. Chlorinated and other VOCs, which are often studied and reported in scientific publications due to their related health concerns, were selected from the data mass and are presented in Table 2c. These chlorinated VOCs were detected very rarely in the ten-year VOC data. The frequency of aromatic styrene was also fairly low. The P99 values of carbon tetrachloride, chloroform, 1,4-dichlorobenzene, and trichloroethene were below the limit of quantitation. The P99 values for tetrachloroethene (0.4 µg/m^3^) and styrene (1.0 µg/m^3^) in turn were far below their RW I and EU-LCI values. It should be noted that Tenax TA has a rather low breakthrough volume for halogenated compounds, such as carbon tetrachloride, trichloroethene, and chloroform, so their concentrations are approximate.

### 3.2. Differences between Indoor Environments

Table 3a shows the differences between the four studied indoor environment types in terms of the frequency and P95 values of the 42 most common VOCs and the TVOC sum variable. Table 3b presents the corresponding information on formaldehyde. The comparison of environments was based on P95 values instead of medians or other lower percentiles because medians of most individual VOCs were below LOQ in all environments, and many of the P90 values were only slightly above LOQ. Geometric or arithmetic means were also regarded as unfit for this data due to the high percentages of measurement results below LOQ. Median and P90 values are presented as additional information for TVOC and formaldehyde, where they have informative value.

The alkanes heptane, nonane, and octane were detected more frequently in the offices (freq. 17–18%) than in the other studied indoor environments (freq. 10–13%), whereas 2,2,4,6,6-pentamethylheptane was more frequently found in healthcare offices (freq. 16%) than elsewhere (freq. 10%). The P95 values were ≤2 µg/m^3^ for all alkanes in all the environments. The differences between the P95 values in the offices and other environments were minor but mainly statistically significant.

Aromatic VOCs, namely benzene, ethylbenzene, 1,2,4-trimethylbenzene, xylenes (p, m, o), toluene, and benzaldehyde (presented under the aldehyde group), were more frequent in the office environments than in the other studied environments. For most of the aromatic compounds presented, the lowest frequencies were measured in the kindergartens. For example, xylenes (p, m) had a frequency of 69% in the offices and 47% in the kindergartens. Despite notable differences in frequency, the differences between the P95 values of the aromatics in the environments were small. The highest P95 values in the aromatic group were observed for toluene: 4–5 µg/m^3^.

Terpenes 3-carene and α-pinene were more frequent in the kindergartens than in the other environments, whereas limonene was the most frequent in the office environment. The lowest frequencies of terpenes were typically observed in the healthcare office environment. The differences between the P95 values were small although mainly statistically significant between office and other environments. The highest P95 values within the terpene group were observed for α-pinene: 6–9 µg/m^3^.

Alcohols generally had the lowest frequencies in the healthcare offices, but the differences between the environments were small. Alcohol ethers and phenol ethers also had the lowest frequencies in the healthcare offices, but their differences to other environments were more prominent. The only representative of the phenol group, namely phenol, had the lowest frequency in the kindergartens, but the differences between the environments were small. The P95 concentrations of alcohols, phenols, alcohol ethers, and phenol ethers were generally rather similar in all the environments. The biggest difference between the P95 concentrations in the different environments was observed for 2-(2-ethoxyethoxy)ethanol and ranged from 1 µg/m^3^ (healthcare office) to 7 µg/m^3^ (school). The highest P95 concentrations within these VOC groups were observed for 1,2-propanediol: 12–14 µg/m^3^.

Apart from aromatic benzaldehyde, the VOCs in the aldehyde group, namely decanal, hexanal, heptanal, nonanal, octanal, and pentanal, had notably higher frequencies in the kindergartens than in the other environments. The P95 concentrations of aliphatic aldehydes were also systematically slightly higher in the kindergartens than in the other environments. The greatest difference observed in P95 values was for nonanal, with a P95 of 11 µg/m^3^ in the kindergartens and 5–6 µg/m^3^ in the other environments.

The only representative of the ketone group, namely acetophenone, had rather similar frequencies (range 21–25%) and P95 concentrations (range 0.8–1 µg/m^3^) in all the studied environments.

The three carboxylic acids presented under the acids group, namely hexanoic acid, pentanoic acid, and propionic acid, had the highest frequencies in the office environment and the lowest frequencies in the healthcare offices. The P95 values of acids were also slightly lower in the healthcare offices than in the other environments. The largest difference between the P95 values was seen in hexanoic acid, with a P95 value of 5 µg/m^3^ in the healthcare offices and 7–8 µg/m^3^ in the other environments.

As regards esters, healthcare office environment displayed generally the lowest frequencies, whereas the environments with the highest frequencies varied. The kindergarten environment stood out with a substantially higher TXIB frequency (57%) than in any other environments (19–21%). n-butyl acetate also displayed a notably higher frequency (20%) in the kindergartens than in the other environments (11–15%). The differences between the frequencies of the other esters in the environments were small. TXIB had the largest difference in P95 values, with a P95 value of 6 µg/m^3^ in the kindergartens and 2–3 µg/m^3^ in the other environments.

The only representative of Si-compounds, namely decamethylcyclopentasiloxane, displayed rather high frequencies (65–76%) in all the environments. Its P95 concentration ranged from 9 µg/m^3^ in the school environments to 12–13 µg/m^3^ in the other environments.

The total sum of the VOCs was rather similar in the different environments, as demonstrated by the TVOC median, P90, and P95 values in Table 3a.

The frequency of formaldehyde was over 90% in all the studied environments (Table 3b). The P95 values of formaldehyde ranged from 14 µg/m^3^ (school) to 25 µg/m^3^ (office). Similarly, the lowest median and P90 values of formaldehyde were observed in the schools and the highest in the offices.

### 3.3. Trends

**Figure 1:**
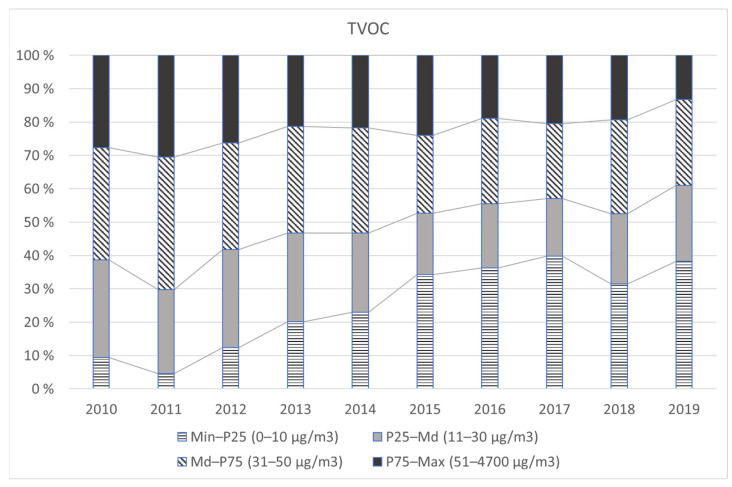
The shift in TVOC distribution in 2010–2019. The four different colour patterns represent the four quarters between the data points of Min, P25, Md, P75, and Max of the ten-year TVOC data (*n* = 9789). It can be seen from the graph that the share of the first quarter (Min–P25) increased and that the share of the top quarter (P75–Max) decreased during the study period.

The total concentration of VOCs (TVOC) showed a strongly decreasing trend over the ten-year study period, 2010–2019. The shift in TVOC distribution is graphically presented in Figure 1. Table 4a presents the annual statistics of the 42 most common VOCs and the TVOC sum variable. Table 4b shows the corresponding information on formaldehyde. For each compound, we analysed the trends of both frequency (i.e., the share of quantifiable measurement results) and concentration. Statistically significant (*p* < 0.0001) trends are indicated by downward and upward arrows. The Z values shown below the arrow symbols indicate the direction and steepness of the trend.

The frequency and concentration trends of all four alkanes were clearly decreasing. This means that they have become rarer findings and that their concentration levels decreased during the ten-year study period. The most prominent decreases in frequency were observed for nonane and octane, which had 24% frequency in 2010 but only 4–5% frequency in 2019. The P95 values of the alkanes decreased during the study period although they were already minor (≤1 µg/m^3^) at the beginning of the study period.

Like those of the alkanes, the frequency and concentration trends of aromatic hydrocarbons were either strongly or moderately decreasing. The most prominent decrease in frequency was observed for benzene, which was present in quantifiable concentrations in nearly all the samples (98%) in 2010 but in only half of the samples (49%) in 2019. The frequency of xylenes (p, m) and ethylbenzene also fell considerably, from 84% to 52% and from 47% to 18%, respectively. These decreasing trends were reflected at the higher ends of the distributions, as demonstrated by the gradual decreases in the P95 concentrations of aromatic compounds.

Both the frequency and concentration of terpenes 3-carene and β-pinene showed slightly increasing trends. However, this was not reflected in their P95 values, which remained fairly stable over the years. The terpenes limonene and α-pinene showed no concentration trends, and α-pinene had only a very slightly increasing frequency trend. Interestingly, the P95 values of limonene decreased gradually from 5–6 µg/m^3^ to around 2 µg/m^3^ during the study period although there was no overall concentration or frequency trend.

Apart from 2-ethyl-1-hexanol, the alcohol compounds displayed a slightly increasing frequency trend and either no concentration trend or a slightly increasing concentration trend. In contrast, 2-ethyl-1-hexanol displayed clearly decreasing frequency and concentration trends. The P95 values of alcohols varied annually but no obvious decreases or increases were observed over the ten years.

Phenol displayed moderately increasing frequency and concentration trends. However, its P95 concentration remained rather steady at around 1 µg/m^3^ over the ten years.

Apart from 2-butoxyethanol, alcohol and phenol ethers displayed slightly decreasing frequency and concentration trends or none at all. The P95 concentrations of alcohol and phenol ethers slightly decreased during the study period. The frequency and concentration trends of 2-butoxyethanol were moderately increasing. However, the P95 concentration of 2-butoxyethanol did not increase over the ten years.

All seven aldehydes displayed increasing frequency trends during the study period, whereas the concentration trends varied. Interestingly, benzaldehyde displayed completely opposite frequency and concentration trends. This means that benzaldehyde is increasingly being detected but that its concentration level is decreasing. Despite some aldehydes showing a slightly increasing concentration trend, the P95 concentrations of aldehydes showed either a slightly decreasing trend or remained fairly stable over the ten years.

The ketone compound acetophenone displayed strongly increasing frequency and concentration trends. However, the P95 concentration of acetophenone stayed at ≤ 1 µg/m^3^ throughout the study period.

Hexanoic, pentanoic and propionic acids showed similar trends of increasing frequency and concentration levels. However, the P95 concentrations of acids decreased during the study period.

Esters showed varying trends. 2-(2-Butoxy ethoxy)ethyl acetate displayed slightly decreasing frequency and concentration trends. n-Butyl acetate showed no significant trends. Ethyl acetate, Texanol, and TXIB displayed slightly increasing frequency trends from 11–16% to around 20%. We also found slightly increasing trends in the concentration levels of Texanol and TXIB. At the same time, the P95 values of all esters remained fairly stable over the ten years.

Decamethylcyclopentasiloxane showed a slightly increasing frequency trend, rising from around 60% to over 70%. At the same time, the concentration trend was slightly decreasing, and the P95 values of this Si-compound varied in the range of 7 to 16 µg/m^3^, with no obvious trend.

Table 4b presents the annual statistics for formaldehyde. The frequency of formaldehyde displayed no trend, as it was high, 89–100%, every year. At the same time, the concentration level of formaldehyde showed a decreasing trend. The decrease in concentration was evident in all parts of the distribution, as demonstrated by the fall in the median, P90, and P95 values from 9 to 3 µg/m^3^, from 25 to 9 µg/m^3^, and from 43 to 19 µg/m^3^, respectively.

## 4. Discussion

### 4.1. Indoor Air Concentrations of VOCs and Formaldehyde Are Generally Low and Pose No Health Risks

A large variety of volatile organic compounds originating from both indoor and outdoor sources are present in indoor environments. According to our ten-year VOC data collected in 2010–2019 in Finland, the most frequent VOCs in offices and similar non-industrial indoor environments are benzene, xylenes (p, m), toluene, α-pinene, 1-butanol, 2-ethyl-1-hexanol, 1,2-propanediol, benzaldehyde, hexanal, nonanal, and decamethylcyclopentasiloxane. These compounds were present in concentrations exceeding the respective LOQs (LOQ values varied 0.3–1 µg/m^3^) in more than half of the 9789 samples collected in 2010–2019; i.e., these twelve compounds had a frequency over 50% in the total ten-year VOC data. Typical sources of these common VOCs are motor-vehicle emissions from outdoors (aromatic compounds), wood-based and other interior and construction materials (α-pinene, alcohols, aldehydes), water-dilutable glues and PVC materials (alcohols), detergents, fragrances, cosmetics (aldehydes, siloxanes), and food supplies (aldehydes).

Despite the VOC’s multiple sources, the TVOC levels and the concentrations of single VOCs were generally low in comparison to previously published data on similar indoor environments [2,7,8,17]. However, the maximum values were generally bigger in our dataset than in other datasets, which is probably due to the notably bigger sample size of our study compared to the samples of others. The median, P90, P95, and P99 TVOC values in our ten-year dataset were 30, 90, 137, and 290 µg/m^3^, respectively. This means that in over 99% of cases, TVOC concentration fell well below 400 µg/m^3^, which is the TVOC reference value set by the Finnish Decree on Housing Health 545/2015. These concentration levels also fall into the stage one (out of five) hygienic guide value of ≤0.3 mg/m^3^ set by the UBA for indoor air TVOC [13,14]. The UBA considers this first stage hygienically safe. Furthermore, the TVOC concentration levels displayed a clearly decreasing trend during the ten-year study period. In the last study year, 2019, the median, P90, P95, and P99 TVOC values were 20, 70, 100, and 215 µg/m^3^, respectively.

Although the majority of VOC sampling was performed in buildings with suspected indoor air problems of some kind (not necessarily VOCs), we believe that the VOC levels measured in our study rather accurately represent those of a normal population of offices and similar non-industrial indoor environments in Finland. Considering the very low levels of VOCs measured in our large dataset, it appears obvious that VOCs are not currently a major concern in Finnish public and office buildings.

Unlike VOC measurements, the formaldehyde measurements (belonging to VVOCs) were targeted to buildings where elevated formaldehyde levels were expected. Nevertheless, the measured formaldehyde levels in our ten-year dataset were low in comparison to previously published data on similar indoor environments [2,7,8,17]. It is also noteworthy that the concentration level of formaldehyde displayed a clear decreasing trend during the ten-year period.

None of the RCRs calculated for the 99th percentile of the VOC and formaldehyde measurements exceeded 1 in comparison to the RW I values; the vast majority were far below this. Therefore, according to the current knowledge on their health hazards, the measured concentrations of these individual substances do not pose health risks. Several of the measured maximum values exceeded their respective RW I value, and a few also exceeded their RW II value. Such measurements originated from individual sampling locations, which should be further investigated.

Many VOCs can be smelled at low concentrations; their odours can be perceived as unpleasant and thus cause nuisance. However, they do not necessarily indicate a health risk, as odour thresholds can be considerably lower than the concentration levels that induce adverse health effects [18].

It should be noted that the occurrence of VOC mixtures was not studied in the dataset, and thus, the health risk assessment does not cover possible mixture effects. These could be relevant particularly for compounds that induce equivalent health hazards via the same action mechanism and would be an interesting topic for further research. However, considering the overall low levels of VOCs in the dataset in comparison to their respective health-based reference values, no immediate concern related to mixture effects is evident from these data.

### 4.2. Differences between VOC Patterns in Different Types of Indoor Environments Have No Practical Relevance

We inspected differences between offices, schools, kindergartens, and healthcare offices and found that these environments differed in terms of frequencies of several VOCs, whereas the differences between the P95 values were typically small. The office environment had a higher frequency of alkanes, aromatic hydrocarbons, and acids than the other environments. The kindergarten environment had a higher frequency of aldehydes, certain terpenes (α-pinene, 3-carene) and certain esters (TXIB, n-butyl acetate). The differences between the measured VOC patterns in these environments probably reflect the differences between their typical distances from busy roads, their typical activities, and the use of construction and interior materials, cleaning products, and utility articles.

Unlike VOCs, formaldehyde showed similar frequency, over 90%, in all the studied environments, whereas the concentration of formaldehyde ranged in a relatively large scale. The lowest median, P90, and P95 concentration values of formaldehyde were observed in the schools and the highest in the offices. As formaldehyde has a multitude of possible emission sources, e.g., traffic, construction materials, furniture, textiles, cosmetics, wood burning, cooking and oxidative reactions, it is difficult to try to explain the observed differences.

Despite the differences between VOC and formaldehyde patterns in these environments, all the measured concentrations were low in comparison with their respective health-based reference values, and therefore, we do not expect these differences to have any practical relevance for health outcomes.

### 4.3. Interpretation of VOC Trends Involves Uncertainties

The total VOC concentration, TVOC, calculated from each VOC sample in our dataset, showed a clearly decreasing trend during the study period, 2010–2019. Both increasing and decreasing trends were observed in individual compounds. Most notably, aromatic compounds and alkanes displayed systematic decreasing trends, whereas several aldehydes and acids showed increasing trends.

We analysed both frequency and concentration trends. The frequency trend reveals whether the frequency value, i.e., the percentage of results above LOQ, is rising or falling. However, the frequency trend does not prove that the concentration level trend is parallel. We found several examples of divergent frequency and concentration trends in our data. Benzaldehyde and decamethylcyclopentasiloxane showed completely opposite trends of increasing frequency and decreasing concentration. In other words, these two compounds were measured more frequently over the years but at the same time the measured concentrations became generally smaller. Formaldehyde showed no frequency trend but a decreasing concentration trend, whereas some VOCs, such as α-pinene and TXIB, showed slightly increasing frequency trend but no concentration trend. In most cases of increasing frequency trend, also the concentration trend was positive, although less prominent (smaller Z). This was true, for example, for several aldehydes, alcohols, and acids. Interestingly, P95 values tended to decrease or remain stable even in situations in which the concentration trend was increasing. In other words, several compounds were detected and quantified more frequently, but a larger share of the measurements were only slightly above the LOQ. One explanation for this phenomenon might be the increased detection and quantification of small peaks in VOC chromatograms due to decreased total VOC concentrations. The ISO 16000-6 standard requires at least two-thirds of the total TVOC area to be identified. This may lead, at low TVOC level, to quantification of very small concentrations that would be neglected with larger TVOC concentrations. Therefore, it is reasonable to view all increasing trends, especially increasing frequency trends, with caution. At the same time, it is important to keep in mind that increased use of textile carpets and epoxy and acrylic resins as well as novel products used in cleaning and personal hygiene might also partially explain some of the increasing trends observed during the study period.

Compared to increasing trends, decreasing trends have less uncertainties due to the increased detection of small peaks discussed above. Aromatic hydrocarbons showed equally strong decreasing frequency and concentration trends during the ten-year study period, 2010–2019. These compounds are mainly linked to fossil fuel combustion [19]. Therefore, we believe that the decreasing trends of aromatic hydrocarbons are mainly connected to better outdoor air quality due to, for example, an ongoing shift towards low-emission vehicles.

The decreasing trends of alkanes and formaldehyde are most likely connected to the increased use of low-emission building and interior materials. This positive development is probably due to the development and application of emission classification systems, the aim of which are to enhance the development and use of low-emission building materials and furniture. In Finland, M1-classification, developed and published for the first time in 1996, sets limit values for the emission of VOCs, formaldehyde, and ammonia as well as criteria for the acceptability of odour [20]. M1-classification is a voluntary labelling system for manufacturers, importers and exporters of building products. However, public property developers often demand the use of M1-certified products in their projects in Finland, thereby enhancing the development and usage of low-emission materials in buildings.

The decreasing frequency and concentration of 2-ethyl-1-hexanol is probably due to the development of non-phthalate plasticizers, which are increasingly being used in flooring materials.

## 5. Conclusions

This study presents a valuable dataset on VOCs and formaldehyde from an exceptionally large number of samples collected from offices and similar indoor environments over a decade in Finland. The data showed that indoor air concentrations of VOCs and formaldehyde in these environments are generally low and pose no health risks. The differences between VOC patterns in office, school, kindergarten, and healthcare office environments have no practical relevance to health outcomes. TVOC concentration showed a clear decreasing trend over the ten-year study period.

## Figures and Tables

**Table:** (**a**) VOC samples.

Year	Office	School	Kindergarten	Healthcare Office
**2010**	384	253	52	119
**2011**	416	310	68	182
**2012**	333	298	67	295
**2013**	385	309	69	190
**2014**	447	344	102	105
**2015**	313	163	42	99
**2016**	324	489	81	195
**2017**	379	548	87	139
**2018**	446	449	90	144
**2019**	445	420	69	139

**Table:** (**b**) Formaldehyde samples.

Year	Office	School	Kindergarten	Healthcare Office
**2010**	19	14	5	6
**2011**	47	37	7	14
**2012**	72	37	5	26
**2013**	35	42	5	28
**2014**	58	95	6	7
**2015**	73	79	9	19
**2016**	38	182	10	10
**2017**	57	131	10	25
**2018**	77	208	5	39
**2019**	45	113	6	10

**Table:** (**a**) Statistics of 42 most frequently detected VOCs in air samples collected from Finnish office and similar non-industrial indoor environments in 2010–2019. Bottom line presents TVOC sum variable statistics. VOCs were sampled and analysed in accordance with ISO 16000-6:2011.

Group	Analyte	CAS	All Samples *n* = 9789 (From Offices, 3872; Schools, 3583; Kindergartens, 727; and Healthcare Offices, 1607 Samples)						Health-Based Reference Values	
			Frequency in Samples 2010–2019 (>LOQ)	Md (µg/m^3^)	P90 (µg/m^3^)	P95 (µg/m^3^)	P99 (µg/m^3^)	Max (µg/m^3^)	RW I/II (µg/m^3^)	EU-LCI (µg/m^3^)
**Alkanes** LOQ = 0.3–0.5 µg/m^3^	Heptane	142-82-5	15%	<LOQ	0.5	0.9	3.0	110	–	15,000
	Nonane	111-84-2	13%	<LOQ	0.4	0.7	1.0	35	–	–
	Octane	111-65-9	13%	<LOQ	0.4	0.6	1.0	12	–	–
	2,2,4,6,6-Pentamethylheptane	13475-82-6	11%	<LOQ	0.4	1.0	6.0	260	–	–
**Aromatic hydrocarbons** LOQ = 0.5 µg/m^3^	Benzene	71-43-2	65%	0.5	1.0	2.0	3.0	31	– ^1^	–
	Ethylbenzene	100-41-4	23%	<LOQ	0.7	1.0	4.0	380	200/2000 ^1^	850
	1,2,4-Trimethyl-benzene	95-63-6	15%	<LOQ	0.5	0.8	2.0	62	400/4000	450
	Xylenes (p, m)	108-38-3, 106-42-3	60%	0.5	2.0	3.0	12	1110	100/800 ^2^	500 ^1^
	Xylene (o)	95-47-6	27%	<LOQ	0.8	1.0	5.0	370	100/800 ^2^	500 ^3^
	Toluene	108-88-3	81%	0.7	3.0	5.0	17	620	300/3000 ^2^	2900
**Terpenes** LOQ = 0.5 µg/m^3^	3-Carene	498-15-7	32%	<LOQ	2.0	3.0	10	620	200/2000	1500
	Limonene	138-86-3, 5989-27-5, 5989-54-8	25%	<LOQ	1.0	3.0	14	1020	1000/10,000	5000
	α-Pinene	80-56-8	64%	0.7	4.0	8.0	24	250	200/2000	2500
	β-Pinene	127-91-3	10%	<LOQ	<LOQ	0.7	2.0	16	200/2000	1400
**Alcohols** LOQ = 0.5 µg/m^3^ except for 1,2-propanediol 0.8–1 µg/m^3^	Benzyl alcohol	100-51-6	19%	<LOQ	0.9	2.0	13	170	400/4000	440
	1-Butanol	71-36-3	73%	0.7	3.0	5.0	13	790	700/2000	3000
	2-Ethyl-1-hexanol	104-76-7	64%	0.6	3.0	7.0	21	230	100/1000 ^1^	300
	2-Methyl-1-propanol	78-83-1	21%	<LOQ	0.8	1.0	4.0	180	–	11,000
	1,2-Propanediol	57-55-6	56%	0.8	7.0	13	45	720	60/600	2100
**Phenols** LOQ = 0.5 µg/m^3^	Phenol	108-95-2	20%	<LOQ	0.8	1.0	4.0	40	20/200	70
**Alcohol and phenol ethers** LOQ = 0.5–1 µg/m^3^	2-(2-Butoxy ethoxy)ethanol	112-34-5	17%	<LOQ	2.0	3.0	11	97	400/2000 ^4^	350
	2-Butoxyethanol	111-76-2	19%	<LOQ	0.8	2.0	8.0	140	100/1000	1600
	2-(2-Ethoxyethoxy) ethanol	111-90-0	21%	<LOQ	2.0	4.0	25	730	700/2000 ^4^	350
	2-Phenoxyethanol	122-99-6	22%	<LOQ	0.9	2.0	5.0	110	30/100	60
	1-Methoxy-2-propanol	107-98-2	14%	<LOQ	0.7	1.0	6.0	370	1000/10,000	7900
**Aldehydes** LOQ = 0.5 µg/m^3^	Benzaldehyde	100-52-7	78%	1.0	2.0	3.0	5.0	76	20/200 ^4^	–
	Decanal	112-31-2	62%	0.7	2.0	3.0	5.0	19	100/2000	900
	Hexanal	66-25-1	56%	0.6	3.0	5.0	14	310	100/2000	900
	Heptanal	111-71-7	17%	<LOQ	0.6	0.9	2.0	20	100/2000	900
	Nonanal	124-19-6	80%	1.0	4.0	6.0	11	75	100/2000	900
	Octanal	124-13-0	38%	<LOQ	1.0	1.0	3.0	36	100/2000	900
	Pentanal	110-62-3	32%	<LOQ	1.0	2.0	4.0	96	100/2000	800
**Ketones** LOQ = 0.5 µg/m^3^	Acetophenone	98-86-2	23%	<LOQ	0.7	0.9	2.0	25	–	490
**Acids** LOQ = 0.5–1 µg/m^3^	Hexanoic acid	142-62-1	39%	<LOQ	5.0	7.0	14	330	–	2100
	Pentanoic acid	109-52-4	12%	<LOQ	0.6	2.0	4.0	98	–	2100
	Propinoic acid	79-09-4	19%	<LOQ	2.0	3.0	8.0	110	–	1500
**Esters** LOQ = 0.3–0.5 µg/m^3^	n-Butyl acetate	123-86-4	14%	<LOQ	0.6	1.0	5.0	150	–	4800
	2-(2-Butoxy ethoxy)ethyl acetate	124-17-4	10%	<LOQ	0.3	1.0	6.0	72	–	850
	Ethyl acetate	141-78-6	14%	<LOQ	0.7	2.0	8.0	450	600/6000	–
	Texanol	25265-77-4	23%	<LOQ	1.0	3.0	15	320	–	850
	TXIB	6846-50-0	23%	<LOQ	2.0	3.0	10	100	–	1300
**Si-compounds** LOQ = 0.5 µg/m^3^	Decamethylcyclo-pentasiloxane	541-02-6	72%	1.0	6.0	11	33	680	100/1000	pending
**TVOC**			100%	30	90	137	290	4700	–	–

**Table:** (**b**) Statistics of formaldehyde in air samples collected from Finnish office and similar non-industrial indoor environments in 2010–2019. Formaldehyde was sampled and analysed in accordance with ISO 16000-3:2011.

Analyte	CAS	All Samples *n* = 1711 (From Offices, 521; Schools, 938; Kindergartens, 68; and Healthcare Offices, 184 Samples)						Health-Based Reference Values	
		Frequency in Samples 2010–2019 (>LOQ)	Md (µg/m^3^)	P90 (µg/m^3^)	P95 (µg/m^3^)	P99 (µg/m^3^)	Max (µg/m^3^)	RW I/II (µg/m^3^)	EU-LCI (µg/m^3^)
**Formaldehyde** LOQ = 1 µg/m^3^	50-00-0	94%	3.8	12	18	46	88	100/-	100

**Table:** (**c**) Statistics of selected VOCs with frequency of <10% in ten-year VOC data collected from Finnish office and similar non-industrial indoor environments in 2010–2019. VOCs were sampled and analysed in accordance with ISO 16000-6:2011.

		All Samples *n* = 9789 (From Offices, 3872; Schools, 3583; Kindergartens, 727; and Healthcare Offices, 1607 Samples)			Health-Based Reference Values	
Analyte	CAS	Frequency in Samples 2010–2019 (>LOQ)	P99 (µg/m^3^)	Max (µg/m^3^)	RW I/II (µg/m^3^)	EU-LCI (µg/m^3^)
Carbon tetrachloride	56-23-5	1.0%	<LOQ	12	–	pending
Chloroform	67-66-3	0.4%	<LOQ	9	–	–
1,4-Dichlorobenzene	106-46-7	0.03%	<LOQ	0.7	–	150
Trichloroethene	79-01-6	0.4%	<LOQ	10	– ^5^	–
Tetrachloroethene	127-18-4	1.1%	0.4	170	100/1000	80
Styrene	100-42-5	5.7%	1.0	18	30/300	250

^1^ Risk-related guide values for carcinogenic substances in indoor air: benzene preliminary value 4.5 µg/m^3^ [14]; ^2^ as total guide values C7–C8 alkylbenzenes [14]; ^3^ LCI value of 500 µg/m^3^ is common for all three xylene isomers (o, m, p) and their mixtures [15]; ^4^ preliminary value [14]. ^5^ Risk-related guide values for carcinogenic substances in indoor air: trichloroethene 20 µg/m^3^ [14].

**Table:** (**a**) Comparison of office environment (shaded with gray) with school, kindergarten, and healthcare office environments in terms of frequency and P95 values of the 42 most frequently detected VOCs. Bottom line presents TVOC sum variable statistics.

Group	Analyte	CAS		Office *n* = 3872	School *n* = 3583	Kindergarten *n* = 727	Healthcare Office *n* = 1607
**Alkanes** LOQ = 0.3–0.5 µg/m^3^	Heptane	142-82-5	Frequency	18%	13% *	11% *	12% *
			P95 (µg/m^3^)	1.0	0.8 *	0.7 *	0.9 *
	Nonane	111-84-2	Frequency	17%	11% *	6% *	11% *
			P95 (µg/m^3^)	0.8	0.6 *	0.5 *	0.6 *
	Octane	111-65-9	Frequency	17%	10% *	12% *	10% *
			P95 (µg/m^3^)	0.7	0.5 *	0.6 *	0.5 *
	2,2,4,6,6-Pentamethylheptane	13475-82-6	Frequency	10%	10%	10%	16% *
			P95 (µg/m^3^)	1.0	1.0	1.0	2.0 *
**Aromatic hydrocarbons** LOQ = 0.5 µg/m^3^	Benzene	71-43-2	Frequency	68%	63% *	67%	59% *
			P95 (µg/m^3^)	2.0	2.0	2.0	1.6 *
	Ethylbenzene	100-41-4	Frequency	28%	22% *	18% *	18% *
			P95 (µg/m^3^)	1.0	1.0	0.8 *	1.0
	1,2,4-Trimethylbenzene	95-63-6	Frequency	18%	14% *	9% *	12% *
			P95 (µg/m^3^)	0.8	0.8	0.6 *	0.9 *
	Xylenes (p, m)	108-38-3, 106-42-3	Frequency	69%	55% *	47% *	55% *
			P95 (µg/m^3^)	3.0	4.0 *	2.0 *	3.0
	Xylene (o)	95-47-6	Frequency	32%	25% *	18% *	22% *
			P95 (µg/m^3^)	1.0	2.0 *	0.9 *	1.0
	Toluene	108-88-3	Frequency	87%	77% *	79% *	79% *
			P95 (µg/m^3^)	5.0	4.0 *	5.0	4.0 *
**Terpenes** LOQ = 0.5 µg/m^3^	3-Carene	498-15-7	Frequency	31%	33%	42% *	28% *
			P95 (µg/m^3^)	3.0	4.0 *	3.0	2.0 *
	Limonene	138-86-3, 5989-27-5, 5989-54-8	Frequency	33%	19% *	24% *	20% *
			P95 (µg/m^3^)	3.4	2.0 *	3.0 *	2.0 *
	α-Pinene	80-56-8	Frequency	65%	62% *	76% *	60% *
			P95 (µg/m^3^)	8.0	9.0 *	7.0 *	6.0 *
	β-Pinene	127-91-3	Frequency	10%	10%	10%	8% *
			P95 (µg/m^3^)	0.7	0.7	0.7 *	0.5 *
**Alcohols** LOQ = 0.5 µg/m^3^ except for 1,2-propanediol 0.8–1 µg/m^3^	Benzyl alcohol	100-51-6	Frequency	19%	20%	18%	15% *
			P95 (µg/m^3^)	2.0	3.0 *	1.0 *	1.0 *
	1-Butanol	71-36-3	Frequency	77%	71% *	75%	68% *
			P95 (µg/m^3^)	4.0	5.0 *	5.6 *	5.0 *
	2-Ethyl-1-hexanol	104-76-7	Frequency	65%	62% *	67%	68% *
			P95 (µg/m^3^)	7.0	6.0 *	6.0 *	8.0 *
	2-Methyl-1-propanol	78-83-1	Frequency	24%	20% *	20% *	18% *
			P95 (µg/m^3^)	1.0	1.0	2.0 *	1.0
	1,2-Propanediol	57-55-6	Frequency	60%	51% *	63%	53% *
			P95 (µg/m^3^)	13	12 *	14 *	13
**Phenols** LOQ = 0.5 µg/m^3^	Phenol	108-95-2	Frequency	22%	18% *	17% *	21%
			P95 (µg/m^3^)	1.0	1.0	1.0	1.0
**Alcohol and phenol ethers** LOQ = 0.5–1 µg/m^3^	2-(2-Butoxy ethoxy)ethanol	112-34-5	Frequency	18%	18%	16%	14% *
			P95 (µg/m^3^)	3.0	4.0 *	3.0	3.0
	2-Butoxyethanol	111-76-2	Frequency	20%	19%	19%	12% *
			P95 (µg/m^3^)	2.0	2.0	2.0	1.0 *
	2-(2-Ethoxyethoxy)ethanol	111-90-0	Frequency	21%	24% *	23%	10% *
			P95 (µg/m^3^)	4.0	7.0 *	5.0 *	1.0 *
	2-Phenoxyethanol	122-99-6	Frequency	22%	21%	24%	21%
			P95 (µg/m^3^)	1.0	2.0 *	2.0 *	2.0 *
	1-Methoxy-2-propanol	107-98-2	Frequency	14%	17% *	15%	9% *
			P95 (µg/m^3^)	1.0	2.0 *	2.0 *	1.0
**Aldehydes** LOQ = 0.5 µg/m^3^	Benzaldehyde	100-52-7	Frequency	80%	77% *	78%	77% *
			P95 (µg/m^3^)	3.0	3.0	3.0	2.0 *
	Decanal	112-31-2	Frequency	60%	61%	71% *	66% *
			P95 (µg/m^3^)	3.0	3.0	4.0 *	4.0 *
	Hexanal	66-25-1	Frequency	54%	55%	79% *	50% *
			P95 (µg/m^3^)	5.0	6.0 *	8.0 *	4.0 *
	Heptanal	111-71-7	Frequency	16%	17%	31% *	13% *
			P95 (µg/m^3^)	0.8	0.9 *	1.0 *	0.7 *
	Nonanal	124-19-6	Frequency	77%	80% *	93% *	81% *
			P95 (µg/m^3^)	5.0	6.0 *	11 *	5.0
	Octanal	124-13-0	Frequency	39%	36% *	49% *	34% *
			P95 (µg/m^3^)	1.0	1.0	2.0 *	1.0
	Pentanal	110-62-3	Frequency	33%	30% *	44% *	25% *
			P95 (µg/m^3^)	2.0	2.0	3.0 *	1.0 *
**Ketones** LOQ = 0.5 µg/m^3^	Acetophenone	98-86-2	Frequency	21%	25% *	23%	24% *
			P95 (µg/m^3^)	0.8	0.9 *	0.8 *	1.0 *
**Acids** LOQ = 0.5–1 µg/m^3^	Hexanoic acid	142-62-1	Frequency	44%	38% *	43%	31% *
			P95 (µg/m^3^)	8.0	7.0 *	8.0	5.0 *
	Pentanoic acid	109-52-4	Frequency	15%	11% *	13%	8% *
			P95 (µg/m^3^)	2.0	1.0 *	2.0	0.8 *
	Propinoic acid	79-09-4	Frequency	23%	18% *	20%	12% *
			P95 (µg/m^3^)	4.0	3.0 *	4.0	2.0 *
**Esters** LOQ = 0.3–0.5 µg/m^3^	n-Butyl acetate	123-86-4	Frequency	15%	13% *	20% *	11% *
			P95 (µg/m^3^)	1.0	1.0	2.0 *	0.9 *
	2-(2-Butoxy ethoxy)ethyl acetate	124-17-4	Frequency	12%	9% *	11%	8% *
			P95 (µg/m^3^)	1.0	1.0	1.0	0.8 *
	Ethyl acetate	141-78-6	Frequency	16%	13% *	14%	14%
			P95 (µg/m^3^)	2.0	1.0 *	2.0	2.0
	Texanol	25265-77-4	Frequency	22%	26% *	24%	17% *
			P95 (µg/m^3^)	3.0	5.0 *	3.0	2.0 *
	TXIB	6846-50-0	Frequency	21%	20%	57% *	19%
			P95 (µg/m^3^)	3.0	2.0 *	6.0 *	3.0
**Si-compounds** LOQ = 0.5 µg/m^3^	Decamethylcyclo-pentasiloxane	541-02-6	Frequency	76%	65% *	70% *	76%
			P95 (µg/m^3^)	13	9.0 *	12 *	12 *
**TVOC**			Frequency	100%	100%	100%	100%
			Md (µg/m^3^)	30	20 *	30	23 *
			P90 (µg/m^3^)	90	90	100 *	90
			P95 (µg/m^3^)	140	130 *	136 *	130 *

**Table:** (**b**) Comparison of office environment to school, kindergarten, and healthcare office environments with regard to frequency and percentile values of formaldehyde.

Analyte	CAS		Office *n* = 521	School *n* = 938	Kindergarten *n* = 68	Healthcare Office *n* = 184
**Formaldehyde** **LOQ = 1 µg/m^3^**	50-00-0	Frequency	95%	93%	96%	91%
		Md (µg/m^3^)	5	3 *	4	4
		P90 (µg/m^3^)	18	10 *	14 *	13 *
		P95 (µg/m^3^)	25	14 *	20 *	17 *

**Table:** (**a**) Annual frequency and P95 values of the 42 most common VOCs during the ten-year study period 2010–2019. For TVOC presented on the bottom line, median, and P90 values are also shown.

Group	Analyte CAS	Trend (freq.) Z	Trend (conc.) Z	Year	2010 *n* = 807	2011 *n* = 971	2012 *n* = 975	2013 *n* = 977	2014 *n* = 995	2015 *n* = 620	2016 *n* = 1089	2017 *n* = 1153	2018 *n* = 1129	2019 *n* = 1073
**Alkanes**	Heptane 142-82-5	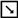	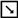	Frequency	20%	20%	22%	16%	12%	13%	12%	9%	11%	13%
		−10	−10	P95 (µg/m^3^)	1.0	1.0	2.0	1.0	0.8	0.8	0.6	0.5	0.6	0.7
	Nonane 111-84-2	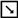	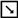	Frequency	24%	25%	23%	20%	15%	9%	5%	3%	6%	5%
		−23	−23	P95 (µg/m^3^)	0.8	0.8	0.9	1.0	0.8	0.6	0.4	<LOQ	0.3	<LOQ
	Octane 111-65-9	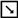	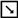	Frequency	24%	32%	24%	15%	14%	5%	4%	3%	5%	4%
		−26	−26	P95 (µg/m^3^)	0.8	0.8	0.8	0.7	0.7	0.4	<LOQ	<LOQ	<LOQ	<LOQ
	2,2,4,6,6-Pentamethylhep-tane 13475-82-6	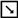	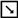	Frequency	13%	21%	20%	19%	9%	12%	8%	5%	6%	5%
		−16	−16	P95 (µg/m^3^)	1.0	4.0	2.0	2.0	0.7	0.7	0.6	<LOQ	0.4	0.4
**Aromatic hydrocarbons**	Benzene 71-43-2	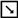	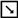	Frequency	98%	89%	73%	70%	59%	52%	61%	49%	56%	49%
		−32	−33	P95 (µg/m^3^)	2.0	2.0	2.0	1.0	1.0	1.0	1.0	1.0	1.0	1.0
	Ethylbenzene 100-41-4	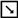	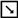	Frequency	47%	36%	22%	21%	17%	24%	20%	14%	20%	18%
		−15	−15	P95 (µg/m^3^)	1.6	1.0	2.0	2.0	0.9	3.0	1.0	0.7	1.0	0.8
	1,2,4-Trimethylbenzene 95-63-6	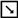	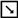	Frequency	31%	23%	13%	15%	9%	14%	17%	9%	11%	12%
		−11	−11	P95 (µg/m^3^)	1.0	0.9	0.7	1.0	0.6	1.0	1.0	0.6	0.6	0.5
	Xylenes (p, m) 106-42-3 108-38-3	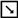	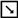	Frequency	84%	73%	66%	54%	53%	60%	58%	49%	60%	52%
		−15	−16	P95 (µg/m^3^)	4.0	4.0	4.0	4.0	3.0	9.0	3.0	2.0	3.0	2.0
	Xylene (o) 95-47-6	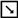	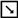	Frequency	48%	38%	25%	24%	21%	26%	26%	17%	26%	22%
		−12	−11	P95 (µg/m^3^)	2.0	1.0	1.0	2.0	1.0	4.0	1.0	1.0	1.0	1.0
	Toluene 108-88-3	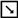	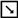	Frequency	98%	90%	89%	83%	77%	76%	78%	71%	82%	75%
		−12	−19	P95 (µg/m^3^)	6.0	4.4	6.0	7.0	4.0	5.0	4.0	3.0	3.0	4.0
**Terpenes**	3-Carene 498-15-7	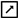	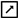	Frequency	26%	24%	23%	29%	26%	37%	44%	39%	39%	36%
		+13	+12	P95 (µg/m^3^)	2.0	2.0	2.0	4.0	3.0	4.0	4.0	3.0	4.0	3.0
	Limonene 138-86-3, 5989-27-5, 5989-54-8	-	-	Frequency	25%	29%	25%	22%	22%	26%	29%	25%	29%	25%
				P95 (µg/m^3^)	5.0	6.0	3.0	2.0	3.0	2.0	2.0	2.0	2.0	2.0
	α-Pinene 80-56-8	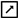	-	Frequency	65%	63%	57%	61%	61%	65%	67%	71%	68%	64%
		+4		P95 (µg/m^3^)	7.0	7.0	7.0	10	8.0	9.0	8.0	9.0	9.0	6.0
	β-Pinene 127-91-3	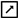	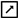	Frequency	7%	5%	7%	11%	8%	12%	14%	11%	13%	12%
		+8	+8	P95 (µg/m^3^)	0.7	0.4	0.5	0.7	0.7	0.7	0.7	0.6	0.8	0.7
**Alcohols**	Benzyl alcohol 100-51-6	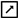	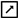	Frequency	17%	11%	16%	19%	23%	22%	24%	20%	19%	19%
		+5	+4	P95 (µg/m^3^)	3.0	1.0	1.2	2.0	3.0	5.0	4.0	2.3	1.0	2.0
	1-Butanol 71-36-3	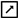	-	Frequency	81%	78%	71%	55%	72%	73%	70%	74%	80%	80%
		+5		P95 (µg/m^3^)	5.0	5.0	4.0	5.0	5.0	5.0	5.0	6.0	4.0	3.0
	2-Ethyl-1-hexanol 104-76-7	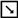	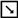	Frequency	80%	67%	63%	60%	74%	59%	64%	60%	60%	59%
		−9	−15	P95 (µg/m^3^)	5.0	9.0	9.0	6.0	4.0	5.0	4.0	7.0	4.0	13
	2-Methyl-1-propanol 78-83-1	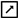	-	Frequency	26%	24%	16%	12%	14%	26%	29%	22%	24%	24%
		+4		P95 (µg/m^3^)	1.6	1.0	1.0	1.0	1.0	2.0	2.0	1.0	2.0	1.0
	1,2-Propanediol 57-55-6	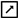	-	Frequency	50%	50%	55%	55%	59%	64%	52%	52%	57%	66%
		+6		P95 (µg/m^3^)	19	12	12	13	15	12	14	9.0	17	9.0
**Phenols**	Phenol 108-95-2	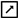	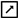	Frequency	18%	11%	11%	10%	12%	11%	34%	27%	26%	30%
		+16	+15	P95 (µg/m^3^)	1.0	1.0	1.0	0.9	1.0	1.0	2.0	2.0	0.9	1.0
**Alcohol and phenol ethers**	2-(2-Butoxy ethoxy)ethanol 112-34-5	-	-	Frequency	14%	18%	17%	18%	21%	20%	19%	12%	17%	17%
				P95 (µg/m^3^)	4.6	4.0	3.0	3.0	4.0	4.0	4.0	3.3	3.0	1.0
	2-Butoxyethanol 111-76-2	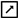	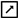	Frequency	8%	12%	13%	14%	16%	27%	26%	29%	23%	17%
		+13	+12	P95 (µg/m^3^)	1.0	1.0	1.0	2.0	1.0	9.0	2.0	2.0	2.0	1.0
	2-(2-Ethoxy ethoxy)ethanol 111-90-0	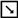	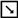	Frequency	16%	22%	24%	27%	25%	23%	20%	13%	17%	17%
		−5	−6	P95 (µg/m^3^)	6.0	4.4	7.0	9.0	5.0	5.0	5.0	2.0	2.0	2.0
	2-Phenoxyethanol 122-99-6	-	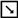	Frequency	13%	22%	26%	29%	22%	27%	25%	17%	19%	18%
			−5	P95 (µg/m^3^)	2.0	2.0	2.0	2.1	2.0	2.0	1.0	0.9	0.9	0.9
	1-Metoxy-2-propanol 107-98-2	-	-	Frequency	13%	15%	13%	16%	15%	16%	16%	13%	15%	13%
				P95 (µg/m^3^)	2.0	2.0	1.0	2.0	1.2	3.0	1.0	1.0	1.0	1.0
**Aldehydes**	Benzaldehyde 100-52-7	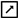	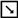	Frequency	67%	58%	65%	76%	77%	86%	87%	87%	85%	90%
		+23	−9	P95 (µg/m^3^)	4.0	3.0	3.0	3.0	3.0	3.0	3.0	3.0	2.0	2.0
	Decanal 112-31-2	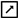	-	Frequency	47%	48%	52%	53%	60%	72%	71%	64%	75%	75%
		+20		P95 (µg/m^3^)	4.0	4.0	4.0	3.0	4.0	3.0	2.0	2.0	2.0	3.0
	Hexanal 66-25-1	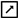	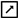	Frequency	49%	61%	50%	54%	52%	43%	55%	54%	66%	68%
		+8	+7	P95 (µg/m^3^)	4.0	4.0	4.0	5.0	6.2	6.0	6.0	7.0	6.0	5.0
	Heptanal 111-71-7	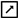	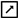	Frequency	12%	20%	13%	14%	15%	12%	10%	13%	25%	31%
		+9	+8	P95 (µg/m^3^)	0.9	1.0	0.8	0.9	0.9	0.8	0.7	0.8	0.8	0.8
	Nonanal 124-19-6	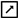	-	Frequency	60%	70%	70%	73%	75%	87%	90%	89%	90%	88%
		+23		P95 (µg/m^3^)	5.0	6.0	7.0	5.1	7.0	6.0	5.0	6.0	5.0	5.0
	Octanal 124-13-0	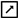	-	Frequency	23%	50%	27%	28%	38%	35%	37%	37%	50%	45%
		+10		P95 (µg/m^3^)	1.0	2.0	2.0	1.0	2.0	1.0	1.0	1.0	1.0	1.0
	Pentanal 110-62-3	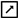	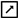	Frequency	23%	27%	18%	20%	16%	39%	40%	38%	49%	44%
		+20	+16	P95 (µg/m^3^)	2.0	2.0	1.0	2.0	2.0	2.0	2.0	2.0	2.0	2.0
**Ketones**	Acetophenone 98-86-2	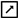	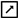	Frequency	2%	8%	18%	31%	28%	15%	26%	25%	36%	31%
		+20	+18	P95 (µg/m^3^)	<LOQ	0.7	1.0	1.0	1.0	0.8	0.8	0.8	0.9	0.8
**Acids**	Hexanoic acid 142-62-1	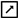	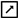	Frequency	21%	31%	22%	23%	28%	47%	51%	56%	51%	55%
		+27	+19	P95 (µg/m^3^)	7.0	8.0	7.0	9.0	11	7.0	6.0	6.3	5.0	5.0
	Pentanoic acid 109-52-4	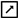	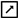	Frequency	8%	7%	3%	11%	10%	17%	17%	20%	16%	14%
		+12	+11	P95 (µg/m^3^)	3.0	2.0	<LOQ	3.0	3.0	1.0	1.0	1.0	1.0	1.0
	Propionic acid 79-09-4	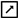	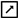	Frequency	10%	12%	6%	12%	6%	20%	21%	22%	28%	46%
		+24	+22	P95 (µg/m^3^)	4.0	4.0	2.0	4.0	2.2	3.0	4.0	3.0	3.0	2.0
**Esters**	n-Butyl acetate 123-86-4	-	-	Frequency	15%	15%	12%	13%	16%	18%	12%	12%	17%	16%
				P95 (µg/m^3^)	2.0	0.9	1.0	1.0	1.0	2.0	1.0	1.0	1.0	0.9
	2-(2-Butoxy ethoxy)ethyl acetate 124-17-4	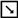	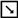	Frequency	7%	10%	12%	10%	13%	12%	13%	9%	6%	6%
		−4	−5	P95 (µg/m^3^)	1.0	2.0	1.0	1.1	2.0	0.8	1.0	0.9	0.5	0.5
	Ethyl acetate 141-78-6	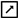	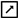	Frequency	12%	15%	15%	9%	8%	16%	13%	14%	18%	23%
		+6	+6	P95 (µg/m^3^)	2.0	2.0	2.0	1.0	1.0	2.0	2.0	2.0	2.0	2.0
	Texanol 25265-77-4	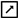	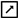	Frequency	11%	17%	19%	22%	28%	28%	25%	27%	31%	20%
		+9	+8	P95 (µg/m^3^)	2.0	3.0	3.0	3.0	4.0	4.0	3.0	3.3	8.0	2.0
	TXIB 6846-50-0	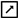	-	Frequency	16%	19%	21%	24%	27%	23%	31%	20%	25%	22%
		+4		P95 (µg/m^3^)	3.0	4.0	2.0	3.0	5.0	4.0	3.0	2.0	2.0	2.0
**Si-compounds**	Decamethylcyclo-pentasiloxane 541-02-6	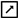	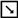	Frequency	57%	69%	75%	70%	70%	74%	73%	73%	76%	74%
		+7	−5	P95 (µg/m^3^)	12	12	11	12	16	16	12	10	9.0	7.0
**TVOC**		-	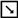	Frequency	100%	100%	100%	100%	100%	100%	100%	100%	100%	100%
			−20	Md (µg/m^3^)	30	40	30	30	30	28	20	20	20	20
				P90 (µg/m^3^)	100	100	100	90	100	130	80	80	80	70
				P95 (µg/m^3^)	150	140	150	150	140	220	125	110	110	100

**Table:** (**b**) Annual statistics of formaldehyde during the ten-year study period 2010–2019.

Analyte CAS	Trend (freq.)	Trend (conc.)	Year	2010 *n* = 44	2011 *n* = 105	2012 *n* = 140	2013 *n* = 110	2014 *n* = 166	2015 *n* = 180	2016 *n* = 240	2017 *n* = 223	2018 *n* = 329	2019 *n* = 174
Formaldehyde 50-00-0	-	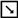 −6	Frequency	100%	91%	96%	92%	89%	93%	94%	91%	95%	99%
			Md (µg/m^3^)	9	10	5	4	3	4	4	3	4	3
			P90 (µg/m^3^)	25	26	15	14	11	11	10	10	12	9
			P95 (µg/m^3^)	43	37	19	23	20	15	14	17	16	19

## Data Availability

The raw data of this study are not publicly available because they are owned by a multitude of laboratory customers.
